# Urban Design Factors Influencing Surface Urban Heat Island in the High-Density City of Guangzhou Based on the Local Climate Zone

**DOI:** 10.3390/s19163459

**Published:** 2019-08-08

**Authors:** Yurong Shi, Yirui Xiang, Yufeng Zhang

**Affiliations:** State Key Laboratory of Subtropical Building Science, Department of Architecture, South China University of Technology, NO. 381 Wushan Road, Guangzhou 510640, China

**Keywords:** land surface temperature, surface urban heat island, social climate zone, retrieval algorithms

## Abstract

Surface urban heat island (SUHI) depicts the deteriorating thermal environment in high-density cities and local climate zone (LCZ) classification provides a universal protocol for SUHI identification. In this study, taking the central urbanized area of Guangzhou in the humid subtropical region of China as the study area, the maps or images of LCZ, land surface temperature, SUHI, and urban design factors were achieved using Landsat satellite data, GIS database, and a series of retrieval and classification algorithms, and the urban design factors influencing SUHI were investigated based on 625 samples of LCZs. The results show that on the 18 September 2016 at the local time of 10:51 a.m., the land surface temperature (LST) varied greatly from 26 °C to 40 °C and the SUHI changed with a wide range of −6 °C to 8 °C in the LCZs of the study area. Seven and five urban design factors influencing the summer daytime SUHI were identified for the two dominant LCZs of LCZs 1–5 (LCZ 1 to LCZ 5) and the mixed LCZ (containing at least three types of LCZs), respectively, in which vegetation cover ratio, floor area ratio, ground emissivity, and complete surface area ratio showed negative correlations and building density showed positive correlations. The summer daytime SUHI prediction models were obtained by using the step-wise multiple linear regression, with the performance of R^2^ of 0.774, RMSE of 0.95 °C, and the d value of 0.91 for the model of LCZs 1–5, and the values of 0.819, 0.81 °C, and 0.94 for the model of the mixed LCZ, indicating that the models can effectively predict the changes of SUHI with LCZs. This study presents a methodology to efficiently achieve a large sample of SUHI and urban design factors of LCZs, and provides information beneficial to the urban designs and regenerations in high-density cities.

## 1. Introduction

Urban heat island (UHI) is the phenomenon that the air temperature in central urbanized areas is warmer than that of the surrounding non-urbanized areas. It is proportional to the degree of urbanization [1,2] and is closely related to urban climatology, thermal environment, and the quality of human life. This phenomenon is significant for high-density cities, for instance, Guangzhou. UHI has been studied by in site measurement, remote sensing retrieval techniques, and modeling [3]. Thermal remote sensors observe the surface urban heat island (SUHI), or more specifically, they “see” the spatial patterns of upwelling thermal radiance received by the remote sensors [4]. The advantage of remotely sensed SUHI is that it can show the spatial variation with a broader region and a higher resolution. Land surface temperature (LST) is the important parameter to analyze urban climatology and is the conventional method to derive the SUHI. It has a direct effect on the air temperature and mean radiant temperature, which directly relate to the thermal environment. Thermal remote sensing of urban surface temperature is a special case of observing LST which varies in response to the surface energy balance [4]. LST has been retrieved from remotely sensed thermal infrared (TIR) images, for instance, using a mono-window algorithm [5,6], or a generalized single-channel method [7] for those of satellites with one TIR band.

As the traditional approach simply uses “rural” and “urban” to define UHI with little attention to the diversity of the urban morphology, the concept of local climate zone (LCZ) [8] was proposed to accurately quantify the relationship between urban morphology and the UHI phenomenon. Primarily, researchers focus on two aspects of studies relating to the LCZ scheme. The first is the LCZ mapping study for one city or a study area, taking LCZ as a lucid way to visualize urban morphology and using satellite images [9,10,11] or GIS data [12,13,14] to form LCZ maps. The second is the thermal properties of LCZs. For instance, Stewart et al. [15], Cardoso and Amorim [16], and Yang et al. [17] studied the heat island magnitude ($\Delta T_{\mathrm{LCZ}\;X-Y}$) using screen-height temperature, which was recorded by sensors installed in various LCZs. Leconte et al. [18], Kotharkar and Bagade [19], and Alexander and Mills [20] compared the air temperature variation in LCZs using mobile measurements. With the distinct definition of the urban landscape and classification hierarchy, LCZ is well-known as a good choice for UHI study. In addition, SUHI is one of the common UHI studies, and the LCZ is a plausible scheme to discriminate LST or SUHI at the local scale. So far, several studies have applied the LCZ to estimate LST or SUHI, for instance, Geletič et al. [21], Cai et al. [10], and Bechtel et al. [22] adopted LST derived from remote sensor images to investigate the thermal environment difference of LCZs in one or multiple cities. Wang et al. [23] compared LST and LCZ changes for different years in Pearl River Delta (China). However, the above studies mainly focused on the magnitude of UHI (SUHI) and thermal performance of LCZs, and very few focused on the main urban design factors influencing UHI (SUHI) in various LCZs.

Oke [1], Oke et al. [24], and Santamouris [2] have reviewed the urban design factors that have influences on UHI, such as canyon radiative geometry, thermal properties of the material of a building or vegetation, anthropogenic heat release, and turbulent transfer. In many SUHI studies [25,26,27], several parameters of sky view factor, impervious surface fraction, vegetation amount, and population were indicated as influencing the formation of SUHI. However, only a few of them were applying the LCZ scheme. As one example, Nassar et al. [25] indicated that proximity to the ocean, sky view factor, height of roughness elements, and building height variations were the major factors governing the zonal SUHI variations in Dubai. They also mentioned that the combined physical variables were more predictive of SUHI variation during summer. Up until now, Giridharan et al. [28,29] established regression models with R^2^ over 0.7, relating urban design variables of surface albedo, average height to floor area ratio, SVF, etc. with UHI. Jusuf and Wong [30] and Jusuf et al. [31] proposed empirical prediction models for an estate level air temperature in Singapore. The above regression models of UHI were all built by using the data of field measurements, with many restrictions on sample size and spatial resolution. The regression models of SUHI could solve these restrictions because LST and many of the urban design factors nowadays can be quickly and easily extracted based on remotely sensed retrieval algorithms [32,33,34] and Geographic Information System (GIS) techniques [35].

There is a dearth of applying the LCZ concept to analyze the influence of urban design factors on SUHI. The objective of this study is to identify the critical urban design factors that influence the SUHI intensity in the high-density city of Guangzhou based on a large sample of LCZs by using a remotely sensed retrieval method and GIS techniques. Moreover, the SUHI prediction models will be proposed for LCZs in the high-density city of Guangzhou. The present study will provide a methodology to efficiently get the information of SUHI at the local scale and the related urban design factors in cities in the largely urbanized zones, and the results are potentially beneficial to urban designs and regenerations in high-density cities.

## 2. Materials and Methods

### 2.1. Study Area

**Figure 1:**
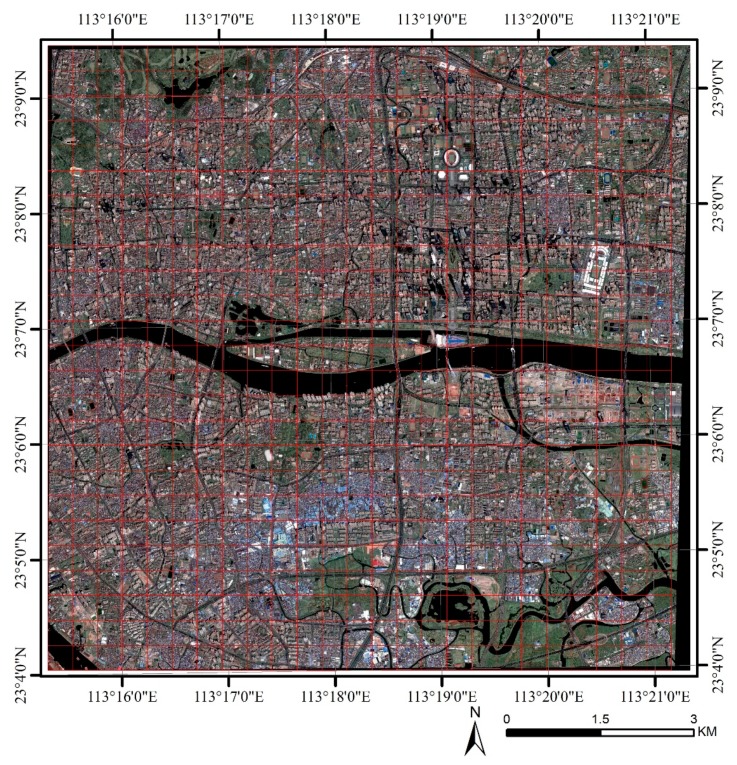
The visible image map with red grids of 400 m × 400 m for the central urbanized area of Guangzhou. This image was obtained by the GeoEye-1 satellite on 22 October 2017.

Guangzhou, located at latitude 23°08′ N and longitude 113°19′ E, is a typical city in the humid subtropical region of China. The total population exceeded 14 million with a population density of 1883 people per km^2^ at the end of 2016, based on data provided by the Statistical Bureau of Guangzhou (http://www.gzstats.gov.cn/). The center of Guangzhou, containing three districts of Tianhe, Yuexiu, and Haizhu, is highly urbanized, as Figure 1 shows. The central urbanized area of Guangzhou was selected as the study area, which was divided into 625 grids of 400 m × 400 m according to the scale of LCZ (see red grids in Figure 1).

In Guangzhou, the long summer is hot and humid with abundant rainfall, and the winter is temperate and short. Guangzhou is in the monsoon region, with prevailing southeast winds in the summer and northern winds in the winter. According to the National Meteorological Information Center (http://data.cma.cn/) averages for over 30 years (from 1981 to 2010), the annual sunshine time is approximately from 1500 to 2000 h; the yearly rainfall is approximately 1801 mm; the monthly mean air temperature is 29.4 °C, and the relative humidity is 75% in July, and those values are 14.1 °C and 68%, respectively, in January in Guangzhou. Overheat is a big risk in summer, and the SUHI during the daytime in summer is of great importance, especially under clear sky conditions.

### 2.2. Local Climate Zone Map

According to the LCZ concept by Stewart and Oke [8], the LCZ system consists of 17 standard LCZs, of which each LCZ has a characteristic urban form with typical geometric and surface cover properties of surface view factor, height-to-width ratio, and surface albedo, etc. Among the 17 LCZs, LCZs 1–10 are built-up areas with differences in building density, height, or type, for example. Compact built-up areas (LCZ 1–3) have high impervious and building surface fraction with various heights of roughness elements, and open built-up areas (LCZ 4–5) have less dense buildings and higher pervious surface fraction. Whereas, LCZs A–G are land cover categories representing a distinct natural landscape, such as trees (LCZ A and LCZ B), bush (LCZ C), low plants (LCZ D), and water (LCZ G).

A LCZ classification map was made for the central Guangzhou using the prevailing method of the World Urban Database and Access Portal Tools (WUDAPT) from the website of http://www.wudapt.org/. There are more than 100 cities worldwide adopting WUDAPT to LCZ studies due to its universal applicability. The procedure was quite lucid. Firstly, eight Landsat 8 images of Guangzhou (Table 1) that meet the criteria of cloudless were downloaded from the USGS website of https://earthexplorer.usgs.gov/. Then, these images were re-sampled from their original 30 m resolution to 100 m resolution in the System for Automated Geoscientific Analyses (SAGA) software to fit the local scale. After that, the training areas of 12 types of LCZs (including 8 built-up LCZs and 4 land cover LCZs) in Guangzhou were digitized through Google Earth and, finally, based on the Landsat 8 images and the training samples, the random forest classifier was adopted to generate the LCZ map of Guangzhou in the SAGA.

Each LCZ has a minimum radius of 200 m (i.e., a diameter of 400 m) to match the local scale in urban climate study [8] and, accordingly, the study area was divided into 625 grids of 400 m × 400 m (Figure 1). The SUHI was calculated by the LST difference between the reference point and each LCZ, in which the mean LST value of LCZ D (${\mathrm{LST}\;D}_{\mathrm{mean}}$) for the central urbanized Guangzhou was chosen as the LST of the reference point because LCZ D represents a traditional non-urbanized area [15,17]. The SUHI could accordingly be expressed as $\Delta T_{\mathrm{LCZ}\;X-D}$, where X means either LCZ, a positive $\Delta T_{\mathrm{LCZ}\;X-D}$ means urban heat island, and a negative $\Delta T_{\mathrm{LCZ}\;X-D}$ means urban cool island.

### 2.3. Remotely Sensed LST

The Landsat 8 satellite data was used for LST retrievals in this study. The image on the date of 18 September 2016 at coordinated universal time (UTC) 2:51 a.m. or the local time of 10:51 a.m. was chosen, which was a recent summer daytime with clear sky conditions. There are two thermal infrared Bands for Landsat 8 TIRS (thermal infrared sensor). Band 10 was used in this study because Band 11 showed the uncertainty of calibration by the USGS. The images from Landsat 8 OLI (Operational Land Imager) in the spatial resolution of 30 m and Landsat 8 TIRS in the spatial resolution of 100 m were obtained.

LST ($T_{s}$) was derived by using the improved mono-window algorithm [6] with the cloud-free pixels from the thermal infrared remote sensing data of the Landsat satellite. The algorithm was based on the original mono-window algorithm [5], which had been verified as a reasonable LST estimation method by comparing it with the simulated data or in situ measured data in the Israel–Egypt border [5] and Guangzhou [36]. The improved algorithm resulted in developments of the acquisition of an estimate of the parameter of effective mean atmospheric temperature and showed a high accuracy of ~1.4 K for LST retrieval [6]. The mono-window algorithm is shown in Equations (1)–(3):

(1) $$T_{s}\;=\;\left[a_{10}\left(1-C_{10}-D_{10}\right)\;+\;\left[b_{10}\left(1-C_{10}-D_{10}\right){\;+\;C}_{10}{\;+\;D}_{10}\right]T_{10}-D_{10}T_{m}\right]{/C}_{10},$$

(2) $$C_{10}\;=\;\varepsilon \tau ,$$

(3) $$D_{10}\;=\;(1-\tau )[1\;+\;(1-\varepsilon )\;\tau ],$$

where $a_{10}$ and $b_{10}$ are constants, $a_{10}$ = −67.355351, $b_{10}$ = 0.458606 for the temperature range of 0 to 50 °C; $T_{10}$ is the brightness temperature in K. $T_{m}$ is the effective mean atmospheric temperature, ε  is the Ground emissivity, and τ is atmospheric transmittance. $T_{m}$, ε, and τ are the important input parameters for the algorithm.

The retrieval method for ground emissivity is presented in detail in Section 2.4. Atmospheric transmittance is one of the most significant parameters for retrieving LST and is mainly affected by the water vapor content (w). The higher the water vapor content is, the lower the level of atmospheric transmittance. Wang et al. [6] proposed a method that uses the air humidity data from a nearby meteorological station to estimate water vapor content. The method was adopted to calculate the atmospheric transmittance, together with the empirical equation of the tropical model proposed in Qin et al. [5] as follows:

(4) $${\tau \;}_{10}=\{\begin{array}{c} 0.9220-0.0780w,\;0.2\;<\;w\;<\;2.0\\ 1.0222-0.1310w,\;2.0\;<\;w\;<\;5.6 \end{array}.$$

The derived values for water vapor content and atmospheric transmittance were 2.796 g/cm^2^ and 0.65 in this study.

### 2.4. Urban Design Factors

There are a large number of urban design factors affecting SUHI. The present study took the nine variables into account, including surface albedo (α), sky view factor (SVF), vegetation cover ratio ($\lambda_{v}$), floor area ratio (FAR), building density ($\lambda_{b}$), ground emissivity (ε), complete surface area ratio (CSAR), mean building height ($Z_{h}$), and average height to building area ratio (HBDG). The detailed specifications and the retrieval methods for the variables are as follows.

#### 2.4.1. Surface Albedo

Surface albedo is the ratio of upwelling irradiance from the surface that is illuminated by an unattenuated direct beam to the surface to downward irradiance, and is a critical parameter affecting the earth’s climate and is commonly required by global and regional climatic modeling and surface energy balance monitoring [37]. Given the solar elevation and azimuth angles, the thermal properties of land surface materials caused the changes in surface albedo, for instance. The canyon albedo can be increased, and the near-ground air temperature can be decreased by increasing the surface albedo of the walls [38].

Surface albedo was derived from Landsat satellite data. He et al. [32] proposed a unified direct estimation approach to evaluate surface albedo from Landsat MSS (Multi-Spectral Scanner), TM (Thematic Mapper), ETM+ (Enhanced Thematic Mapper Plus), and OLI data. This approach was adopted to obtain the surface albedo in the present study, as the validation results against ground measurements show that it was effective with a RMSE lower than 0.034.

#### 2.4.2. SVF

SVF is a parameter that is often used to describe the effect of building geometry on the radiation exchange in the urban environment [39]. SVF is the ratio of the radiation received by a planar surface from the sky to that received from the entire hemispheric radiating environment [40]. A positive correlation between LST and SVF has been confirmed by Scarano et al. [41,42].

Gál et al. [35] proposed two methods to compute continuous SVF values for a whole study area: vector based and raster based algorithms. The first algorithm was used in this study as the small SVF values can be included in the calculation of the cell average, whereas the latter algorithm overestimates it [35]. The improvements by Gál and Unger [43] on tree-crown were also updated in the algorithm.

#### 2.4.3. Vegetation Cover Ratio

Vegetation cover ratio is an indispensability factor for analyzing the impact of vegetation on SUHI. Increasing the vegetation cover ratio is an efficient way to change the surrounding thermal environment. Vegetation cooled surrounding environments in the daytime during summer due to its shading and evaporation effects, while at night, the air temperature in tree shade was higher than that of the space without shade due to the block of upward long-wave radiation [44,45]. Vegetation cover area was derived by the ArcGIS vectorization based on the high-resolution image (Figure 1) and the GIS database for the central urbanized area of Guangzhou.

#### 2.4.4. Floor Area Ratio and Building Density

Floor area ratio and building density are the indices that urban planners and designers often use in their planning and design. Floor area ratio is the ratio of the whole floor area to the entire land area. Previous studies [38,46] showed that in the summer daytime conditions, the air temperature of the urban canyon increased with the decreasing aspect ratio, because the heat gain increment of the air was greater than the thermal storage. As a smaller aspect ratio corresponds to a smaller floor area ratio, a negative relationship between the floor area ratio and UHI can be inferred. Building density is the ratio of gross building area to the entire land area. Bonafoni and Keeratikasikorn [47] show the positive relationship between building density and LST or SUHI. Building footprints and heights were derived by the ArcGIS vectorization, and then floor area ratio and building density were calculated based on the parameters of building footprints and heights.

#### 2.4.5. Ground Emissivity

Ground emissivity is the ratio of energy emitted from the land surface to that from an ideal blackbody at the same temperature. It affects LST or SUHI through long-wave radiations. It has not been reliably measured [48]. Wang et al. [6] got the average emissivity of representative terrestrial materials for Band 10 and Band 11 of Landsat 8 by using the ASTER spectral database [49], and the results showed that the emissivity of water, vegetation, soil, and building were 0.991, 0.973, 0.966, and 0.962, respectively.

The ground emissivity ($\varepsilon_{\lambda }$) in this study was the narrowband emissivity for Band 10 and Band 11 of Landsat 8. It was retrieved by the widely used NDVI (Normalized Difference Vegetation Index) threshold method [33,34]:

(5) $$\varepsilon_{\lambda }=\{\begin{array}{cc} \varepsilon_{s\lambda },\; & {\mathrm{NDVI}\;<\;\mathrm{NDVI}}_{s}\\ \varepsilon_{v\lambda }P_{v}{\;+\;\varepsilon }_{s\lambda }\left(1-P_{v}\right){\;+\;C}_{\lambda },\; & {\mathrm{NDVI}}_{s}\;\leq \;\mathrm{NDVI}\;\leq {\;\mathrm{NDVI}}_{v}\\ \varepsilon_{v\lambda }P_{v}{\;+\;C}_{\lambda },\; & {\mathrm{NDVI}\;>\;\mathrm{NDVI}}_{v}\; \end{array}$$

where the subscript s and v represent soil and vegetation pixels; $P_{v}$ is the vegetation cover ratio, and was retrieved by the dimidiate pixel model [50,51]; $C_{\lambda }$ is a term that takes into account the cavity effect due to surface roughness; ${\mathrm{NDVI}}_{s}$ and ${\mathrm{NDVI}}_{v}$ values were derived from the NDVI histogram [52].

For an urban area that mainly comprised building and vegetation surfaces, the ground emissivity was determined by replacing $\varepsilon_{s\lambda }$ as the emissivity of building $\varepsilon_{b\lambda }$. When NDVI was smaller than zero (NDVI <  0), the pixel was recognised as water surface.

#### 2.4.6. The Complete Surface Area Ratio

The complete surface area proposed by Voogt and Oke [53] is the total sum of the roof, wall, and ground surface area. The larger the complete surface area is, the lower the SUHI or LST. The reason for this is similar to that of the impact of the floor area ratio. The complete surface area ratio was used in the present study and was refined as the ratio of the complete surface area to the entire land area. The complete surface area ratio was derived based on the GIS database of building footprints and heights.

#### 2.4.7. Mean Building Height and the HBDG

Mean building height is an important parameter to calculate aerodynamic properties of urban areas, like zero-displacement length and roughness length [54]. Also, the HBDG is a good parameter that represents the thermal mass of the environment with the effective distance [28,30]. Mean building height and HBDG were derived based on the parameters of building footprints and heights.

### 2.5. Data Analysis and Modelling

**Figure 2:**
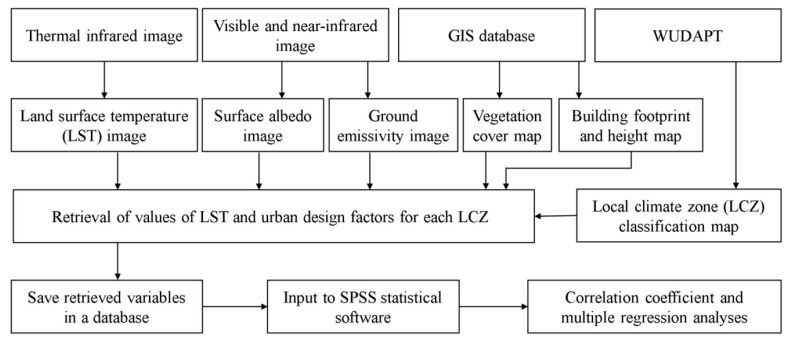
The framework of this study.

The data samples were obtained by combining the data from the LCZ map, LST image, and the urban design factors retrievals. They were saved into a database and put into SPSS statistics software for data analysis. The Pearson correlation analysis and the multiple linear regression were conducted on all data samples to observe the changing tendency of SUHI with various urban design factors and to identify the key factors with significant and high correlations with SUHI. Five-sixths of the samples were then randomly selected for generating the SUHI prediction models by using the step-wise multiple linear regression method, and the remaining one-sixth of the samples were used to validate the models. The criteria of root mean square error (RMSE), index of agreement (d), and coefficient of determination (R^2^) were chosen to reflect the performance of the models. Figure 2 illustrates the details of the data analysis framework in this study.

## 3. Results

### 3.1. LCZ Classification Map

**Figure 3:**
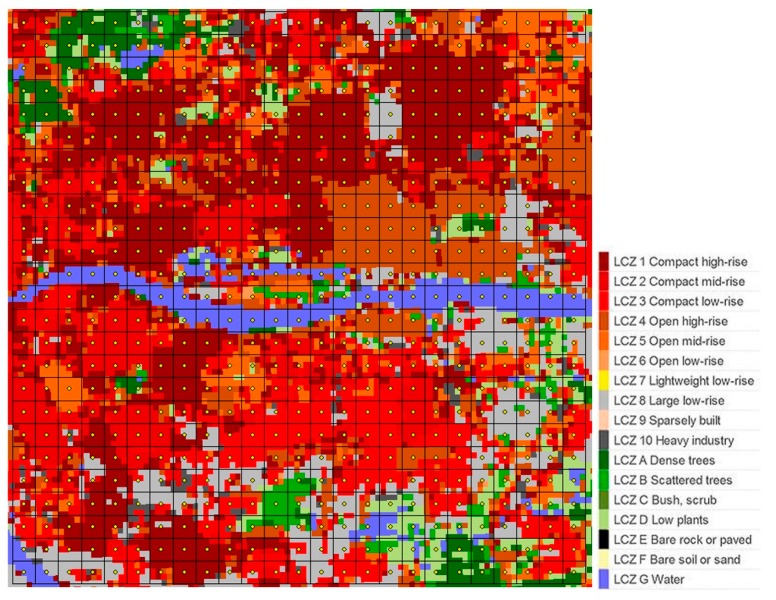
LCZ classification map with grids of 400 m × 400 m for the central urbanized area of Guangzhou.

The LCZ classification map for the central urbanized area of Guangzhou is shown in Figure 3. It demonstrates that the central Guangzhou is a high-density heterogeneous area with 11 different LCZs. A total of 625 samples of LCZs were identified on the map. A confusion matric was calculated to validate the accuracy of the LCZ map by using the randomly selected samples and Google Earth. The overall accuracy was determined to be 79.90%, with a Kappa coefficient of 77.38%, and the accuracies of the built-up areas and the natural land cover were 83.56% and 72.37%, respectively. Compared to those of other high-density regions, such as Yangtze River Delta, China (66.53%) [10], Pearl River Delta (76%) [23], and Hong Kong (58%) [55], the overall accuracy of the LCZ map in the present study is quite acceptable.

**Figure 4:**
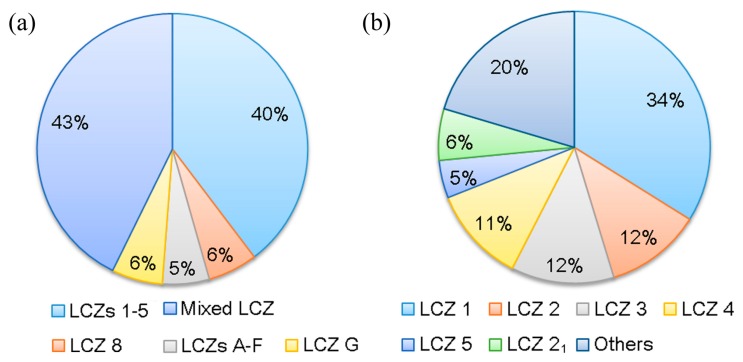
proportion of (**a**) the whole LCZ classification, and (**b**) the built-up LCZs from LCZ 1 to LCZ 5 for the central urbanized area of Guangzhou. “Others” means containing any two types of LCZs 1–5.

The proportion of various types of LCZs was analyzed as shown in Figure 4a. The mixed LCZ, which contains at least three types of LCZs and was not considered in the original paper of LCZ [8], accounts for the largest proportion of 43%. This is in accordance with many other metropolises in China, and the mixed LCZ was therefore treated as the first focus of this study. Besides, LCZ 1 to LCZ 5 (LCZs 1–5) that are built-up areas with open areas scattered sporadically account for a large proportion of 40%. LCZs 1–5 were then taken as the second focus of this study. The detailed proportion of each LCZ in LCZs 1–5 was further investigated, as shown in Figure 4b, indicating that LCZ 1 takes the largest part, followed by the mix of any two types and LCZs 2–4.

### 3.2. Retrievals of LST and Urban Design Factors

**Figure 5:**
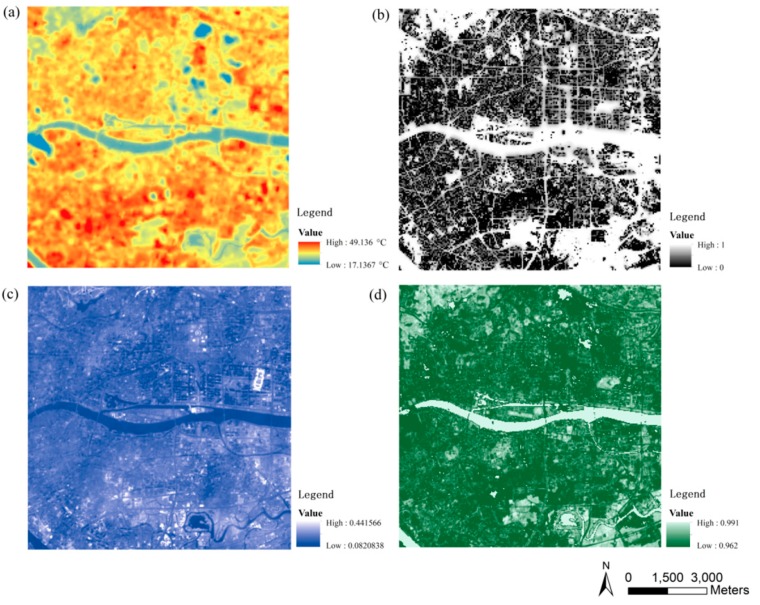
(**a**) Land surface temperature (LST), (**b**) sky view factor, (**c**) surface albedo, and (**d**) ground emissivity retrieval images for the central urbanized area of Guangzhou.

**Figure 6:**
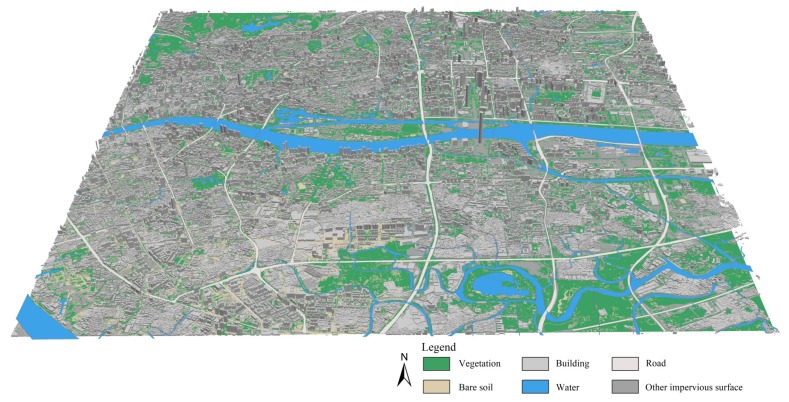
3D land surface cover map for the central urbanized area of Guangzhou.

The retrieved images for LST, SVF, surface albedo, and ground emissivity are shown in Figure 5. The 3D map for land surface cover is shown in Figure 6.

**Figure 7:**
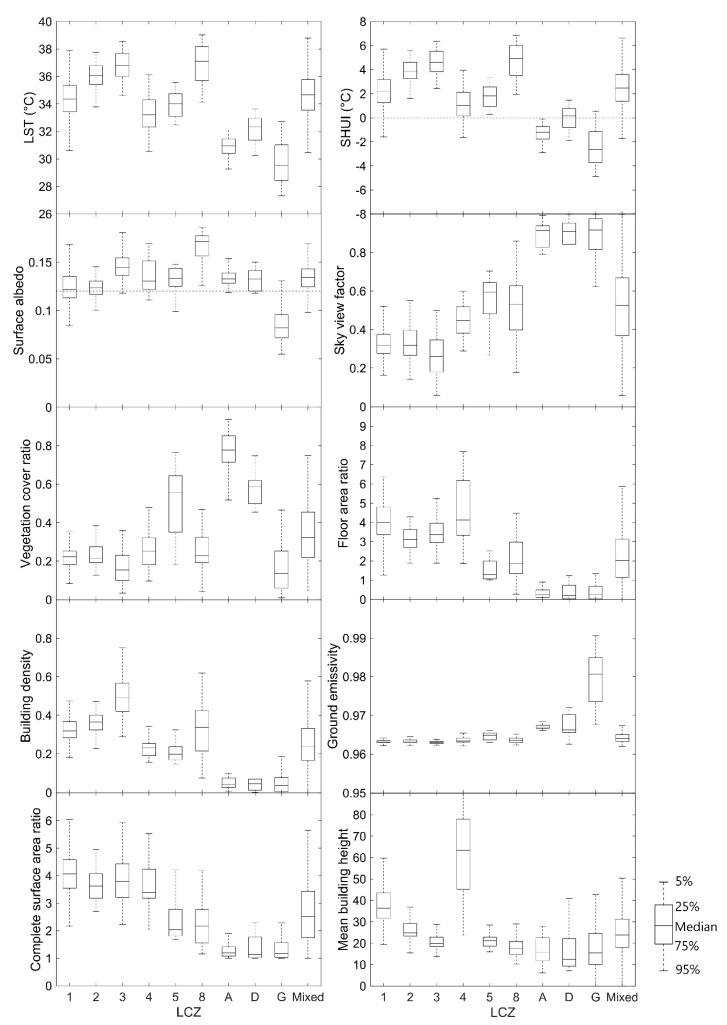
Box plots of LST, surface urban heat island (SUHI), and urban design factors for the LCZs in the central urbanized area of Guangzhou.

Combining Figure 3, Figure 5, and Figure 6, the medians and variations of LST, SUHI, and urban design factors for each LCZ in the central Guangzhou were obtained, as shown in Figure 7. Ten types of LCZ, including 6 built-up LCZs, 3 natural land cover LCZs, and one mixed LCZ, are presented, as the samples for other types are quite small.

On 18 September 2016 at the local time of 10:51 a.m., the LST varied greatly from 26 °C to 40 °C, and the SUHI changed, with a wide range of −6 °C to 8 °C in the LCZs of the study area. The LST and SUHI in various LCZs shows the orders from high to low as LCZ 3 > LCZ 2 > LCZ 1, LCZ 8 > LCZ 5 > LCZ 4, and LCZ D > LCZ A > LCZ G. The orders are in good agreement with the findings by Bechtel et al. [22] on a comparison of 50 cities. All of the median SUHI intensities in the built-up zones showed positive values and negative values for the natural LCZs. The mixed LCZ showed the median between that of the built-up and natural LCZs and the largest variation.

Urban design factors also varied with LCZs, as Figure 7 shows. The surface albedo varied in the range of 0.05 to 0.2. It increased when the built-up areas became more open or the buildings became lower, and it was quite similar for the green natural areas of trees (LCZ A) and plants (LCZ D), while that of water (LCZ G) was much lower. The sky view factor changed greatly from 0 to 1. It was larger in the natural and open built-up areas and, again, the mixed LCZ showed a large variation due to its complexity. The vegetation cover ratio nearly kept the same level of 0.2 for the build-up areas except LCZ 5, and there is no doubt that LCZ A (Dense trees) had the largest values. The two areas of LCZ 1 and 4 with high-rise buildings showed higher floor area ratios (~4), and the two areas of LCZ 3 and 8 with low-rise buildings showed larger building densities (0.5 and 0.3), while the mixed LCZ showed the lower medians of floor area ratio (2) and building density (0.2) with larger variations. The ground emissivity was very similar for all the built-up areas (~0.96), and that of water (0.98) was higher than that of trees and low plants (0.965). The complete surface area ratio varied from 1 to 6 and decreased when the built-up areas became more open or the buildings became lower. The mean building height was largest in LCZ 4 because the Central Business District (CBD) in Guangzhou with many super high-rise buildings was involved in this type of LCZ.

### 3.3. The Correlations between Urban Design Factors and SUHI

Since the multiple linear regression showed that the HBDG had a collinearity problem with the mean building height, with the tolerance smaller than 0.1 and the value of VIF larger than 10, the factor of the HBDG was removed. The remaining eight factors of surface albedo, sky view factor, vegetation cover ratio, floor area ratio, building density, ground emissivity, complete surface area ratio, and mean building height were assumed to play roles in determining SUHI.

In the present study, 221 data samples were obtained for LCZs 1–5 and 241 data samples were obtained for the mixed LCZ. LCZs 1–5 were regular built-up areas with various urban design factors, and useful information for the design of urban built-up areas could be gained by polling all LCZs 1–5 into a data group for analysis. On the other hand, the mixed LCZ were quite complicated and could not be simply treated as built-up or natural land areas. Therefore, the mixed LCZ was treated as another group of data for analysis. It is worth mentioning that 49 data samples with cloud cover were removed from the following analysis because the LST retrieval method is only applicable to the clear sky condition.

#### 3.3.1. Pearson Correlation Analysis

**Figure 8:**
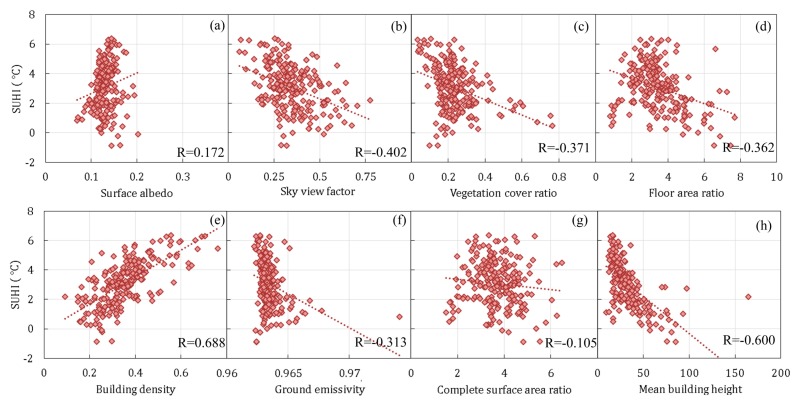
SUHI versus urban design factors of (**a**) surface albedo, (**b**) sky view factor, (**c**) vegetation cover ratio, (**d**) floor area ratio, (**e**) building density, (**f**) ground emissivity, and (**g**) complete surface area ratio, and (**h**) mean building height for LCZs 1–5 in the central urbanized area of Guangzhou.

The Pearson correlation analysis results for LCZs 1–5 are shown in Figure 8. The factors of surface albedo and building density showed positive correlations with the summer daytime SUHI, while other factors showed negative correlations. The changing tendencies of the factors of vegetation cover ratio, floor area ratio, building density, ground emissivity, mean building height, and complete surface area ratio are in good agreement with the previous findings in Section 2.4. The correlation in its absolute value was larger for building density and mean building height, followed by the factors of sky view factor, vegetation cover ratio, floor area ratio, and ground emissivity, while it was less than 0.3 for others.

**Figure 9:**
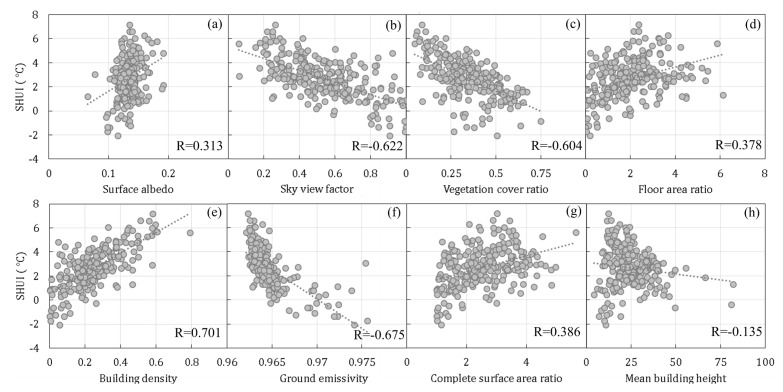
SUHI versus urban design factors of (**a**) surface albedo, (**b**) sky view factor, (**c**) vegetation cover ratio, (**d**) floor area ratio, (**e**) building density, (**f**) ground emissivity, and (**g**) complete surface area ratio, and (**h**) mean building height for the mixed LCZ in the central urbanized area of Guangzhou.

The Pearson correlation analysis results for the mixed LCZ are shown in Figure 9. The factors of surface albedo, floor area ratio, building density, and complete surface area ratio show positive correlations with the summer daytime SUHI, while other factors show negative correlations. The changing tendencies of vegetation cover ratio, building density, ground emissivity, and mean building height are in good agreement with LCZs 1–5 and the previous findings. The correlation in its absolute value is larger for building density and ground emissivity, followed by the factors of sky view factor and vegetation cover ratio, while it is less than 0.4 for others.

Both for LCZs 1–5 and the mixed LCZ, the correlations of surface albedo and sky view factor with the summer daytime SUHI showed opposite signs to those of previous findings. Furthermore, the two factors of floor area ratio and complete surface area ratio showed opposite correlations in LCZs 1–5 and in the mixed LCZ. The above abnormal results indicate that the Pearson correlation analysis may not reveal the actual correlations in the case of multivariate changes.

#### 3.3.2. Partial Correlation Analysis

The multiple regression results for LCZs 1–5 and the mixed LCZ are presented in Table 2. The results of the Pearson correlation analysis are shown together for comparison.

In LCZs 1–5, the factors of sky view factor and building density showed positive partial correlations with the summer daytime SUHI, while other factors showed negative partial correlations. Compared to those of the Pearson correlation, it can be observed that the partial correlations became opposite for the factors of surface albedo and sky view factor. In the mixed LCZ, the factors of building density showed positive partial correlations with the summer daytime SUHI, while other factors showed negative or insignificant partial correlations. The partial correlations were opposite to the Pearson correlations for the factors of floor area ratio and complete surface area ratio.

Compared with the specifications in Section 2.4, it can be found that all the results of the partial correlations are in good agreement with the well-known facts or previous findings, indicating that the partial correlation is more suitable for capturing the actual correlations in the case of multivariate changes. The exclusion of mean building height in the regressions both for LCZs 1–5 and the mixed LCZ was due to their significance levels (*p*) being larger than 0.05. In addition, since sky view factor had collinearities with floor area ratio and *p* > 0.05 for surface albedo in the mixed LCZ, both of them were removed from the regression.

### 3.4. The SUHI Prediction Models and Their Validations

The randomly selected five-sixths of the samples were used for generating the summer daytime SUHI prediction models by using a step-wise multiple linear regression method, in which the factors with an insignificant impact (*p* > 0.05) or with the collinearity problem were removed. Finally, seven factors for LCZs 1–5 and five factors for the mixed LCZ were determined, as shown in Table 3.

According to Table 3, the summer daytime SUHI prediction model for the LCZs 1–5 in the high-density city of Guangzhou was obtained as:

(6) $$\begin{array}{c} \mathrm{SUHI}\;=\;235.605-15.952\alpha \;+\;4.721\mathrm{SVF}-5.281\lambda_{v}-0.373\mathrm{FAR}\;+\;10.988\lambda_{b}-240.539\varepsilon \\ \;\;\;\;-0.447\mathrm{CSAR},\\ R^{2}=\;0.737 \end{array}$$

and accordingly, the summer daytime LST prediction model was obtained as:

(7) $$\begin{array}{c} {\mathrm{LST}=\mathrm{LST}\;D}_{\mathrm{mean}}\;+235.605-15.952\alpha \;+\;4.721\mathrm{SVF}-5.281\lambda_{v}-0.373\mathrm{FAR}\;+\;10.988\lambda_{b}-240.539\varepsilon \\ \;\;\;\;-0.447\mathrm{CSAR},\\ R^{2}=\;0.737. \end{array}$$

The summer daytime SUHI and LST prediction models for the mixed LCZ in the high-density city of Guangzhou were obtained as:

(8) $$\mathrm{SUHI}\;=\;405.985\;-5.749\lambda_{v}-0.303\mathrm{FAR}\;+\;5.889\lambda_{b}-415.054\varepsilon -0.685{\mathrm{CSAR},\;R}^{2}=\;0.790.$$

(9) $$\begin{array}{c} {\mathrm{LST}=\mathrm{LST}\;D}_{\mathrm{mean}}\;+\;405.985-5.749\lambda_{v}-0.303\mathrm{FAR}\;+\;5.889\lambda_{b}-415.054\varepsilon \\ -0.685{\mathrm{CSAR},\;R}^{2}=\;0.790. \end{array}$$

**Figure 10:**
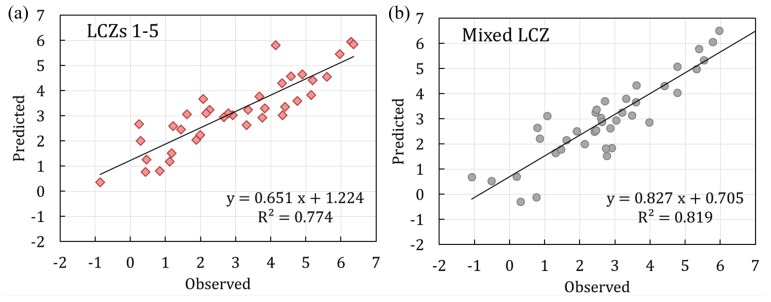
Observed versus predicted SUHI values for (**a**) LCZs 1–5 and (**b**) the mixed LCZ models. The linear regression with solid lines are shown.

The comparisons between the observed and the predicted SUHI values for the remaining one-sixth of the samples of LCZs 1–5 and the mixed LCZ are illuminated in Figure 10. The observed and predicted values had a positive correlation, with R^2^ of 0.774 and 0.819 for the models of LCZs 1–5 and the mixed LCZ, respectively. The RMSE values were obtained as 0.95 °C and 0.81 °C for the LCZs 1–5 and mixed LCZ models, respectively, showing a satisfactory performance. A value of d close to 1.0 indicates that the model prediction approaches the observed variable. The d values of the LCZs 1–5 and mixed LCZ models were determined to be 0.91 and 0.94, respectively, indicating that the prediction models could effectively predict the changes of SUHI with LCZs.

**Figure 11:**
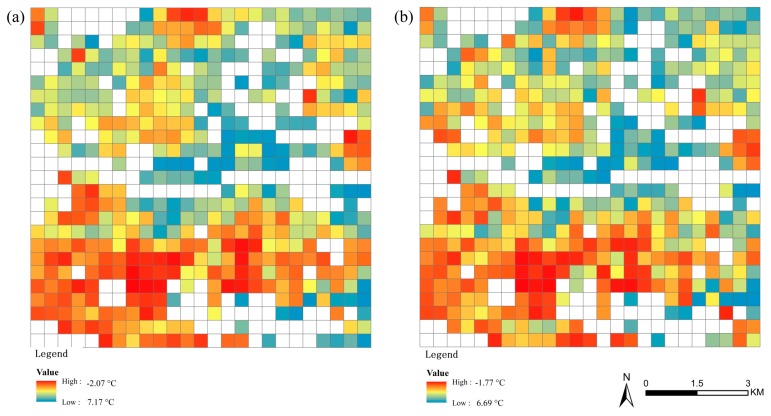
The consistency of (**a**) Observed and (**b**) Predicted SUHI images for both LCZs 1–5 and the mixed LCZ; the white grids are neither LCZs 1–5 nor mixed LCZ.

The consistency between the observed and the predicted SUHI values for both LCZs 1–5 and the mixed LCZ is further observed in Figure 11. It is clearly shown that the surface urban cool and heat islands are distributed consistently in the two images, and the ranges of the observed and the predicted SUHI values are quite close to each other. This confirms that the two prediction models can predict the SUHI distributions very well.

## 4. Discussion

The results of the present study underline the importance of urban design factors in SUHI at the local scale. Consequently, the SUHI intensities are increasing with the higher building density or the larger impervious surface fraction. This result is in good agreement with previous findings. For instance, in the case of Imhoff et al. [26], who indicated that impervious surface area was the primary driver for increases in temperature (explaining 70% of the total variance in LST). Similar results were observed in Clinton and Gong [27], who indicated that the more thermal mass was incorporated into cities, the more the surface differentials would increase.

The SUHI intensities are decreasing with the increasing vegetation cover ratio, mainly because vegetation increases latent heat fluxes in the air through evaporation and transpiration. This result agrees with the previous studies of Clinton and Gong [27] who indicated a negative relationship between SUHI and vegetation. Similarly, floor area ratio and complete surface area ratio have negative influences on SUHI, indicating that the zones with higher building height and more building shading exhibit a lower SUHI. This shares the same result with the finding of Nassar et al. [25], who indicated that building height had an influence on daytime cooling.

This study is one of the few studies that applied the LCZ concept to analyze the influence of urban design factors on SUHI. A methodology is provided for this by achieving the maps or images of LCZ, LST, SUHI, and urban design factors by using Landsat satellite data, the GIS database, and a series of retrieval and classification algorithms. The proposed SUHI prediction models are potentially used by the urban planners and designers to easily capture the impact of urban design factors on SUHI and quickly predict the thermal performance of their urban designs in the decision-making process.

Limitations and prospects are briefly listed below. Only one thermal infrared image in the summer daytime was used in this study. More useful information can be provided by including more images, especially those in the summer nighttime and other seasons, and this study provides an efficient and feasible methodology for doing that. The retrieval of air temperature has been studied by many researchers and several algorithms have been proposed. The urban canopy air temperature and its related UHI are recommended to be studied. Although the multiple linear prediction models are effective and simple to use, especially for the early stage of urban planning and design, the following work is worth doing to improve the model’s performance: (1) to include more potential factors, for instance, the population scale and related anthropogenic heat; (2) to re-construct the urban design factors into indicators by following a clear physical mechanism; (3) to consider the interactions of neighboring LCZs.

## 5. Conclusions

In the present study, taking the central urbanized area of Guangzhou, a high-density metropolis, in the humid subtropical region of China as the study area, the maps or images of LCZ, LST, SUHI, and urban design factors were achieved using Landsat satellite data, the GIS database, and a series of retrieval and classification algorithms, and the urban design factors influencing the summer daytime SUHI were investigated based on a large sample (625 samples) of LCZs. The main conclusions are as follows.

The central urbanized area of Guangzhou is a high-density heterogeneous area with 11 different LCZs, in which the mixed LCZ, which contains at least three types of LCZs, and the built-up LCZs 1–5 are dominant.

On 18 September 2016 at the local time of 10:51 a.m., which was during summer in the daytime with clear sky conditions, the LST varied greatly from 26 °C to 40 °C and the SUHI changed, with a wide range of −6 °C to 8 °C in the LCZs of the study area. For the built-up LCZs, the LST or SUHI became higher when the buildings became lower, together with higher surface albedo and building density and lower floor area ratio and complete surface area ratio; while the LST or SUHI became lower when the area became more open, together with higher surface albedo, SVF and vegetation cover ratio, and lower floor area ratio, building density, and complete surface area ratio.

Seven urban design factors influencing the summer daytime SUHI were identified for LCZs 1–5, in which sky view factor and building density showed positive partial correlations, while the factors of surface albedo, vegetation cover ratio, floor area ratio, ground emissivity, and complete surface area ratio showed negative partial correlations.

Five urban design factors influencing the summer daytime SUHI were identified for the mixed LCZ, in which building density showed positive partial correlations, while the factors of floor area ratio, vegetation cover ratio, ground emissivity, and complete surface area ratio showed negative partial correlations.

The summer daytime SUHI prediction models were obtained using the step-wise multiple linear regression, showing the performance of R^2^ of 0.774, RMSE of 0.95 °C, and the d value of 0.91 for the model of LCZs 1–5, and the values of 0.819, 0.81 °C, and 0.94 for the model of the mixed LCZ, respectively. It is concluded that the prediction models can effectively predict the changes of SUHI with LCZs.

The findings of the present study support a methodology to apply the LCZ scheme in analyzing the influence of urban design factors on SUHI effect and provide SUHI prediction models for urban planners and designers for their climate-sensitive urban designs. Further studies are needed to analyze the seasonal and nocturnal SUHI with more potential influencing factors, and also to consider the interactions between neighboring LCZs.

## Figures and Tables

**Table 1:** Landsat 8 images of Guangzhou for the local climate zone (LCZ) map.

Entity ID	Date
LC81220442013333	29 November 2013
LC81220442013365	31 December 2013
LC81220442014288	15 October 2014
LC81220442015003	3 January 2015
LC81220442015019	19 January 2015
LC81220442015291	18 October 2015
LC81220442016038 LC81220442016342	7 February 2016 7 December 2016

**Table 2:** The Pearson correlation and multiple regression results of SUHI versus urban design factors.

Variables	LCZs 1–5					Mixed LCZ				
	Coefficient				Sig.	Coefficient				Sig.
	B	β	Pearson Correlation	Partial Correlation		B	β	Pearson Correlation	Partial Correlation	
Constant	228.161				0.000	405.509				0.000
α	−15.728	−0.198	0.172	−0.297	0.000	EX	EX	EX	EX	EX
SVF	4.266	0.338	−0.402	0.260	0.000	EX	EX	EX	EX	EX
$\lambda_{v}$	−5.243	−0.375	−0.371	−0.388	0.000	−5.118	−0.458	−0.604	−0.568	0.000
FAR	−0.422	−0.353	−0.362	−0.403	0.000	−0.345	−0.263	0.378	−0.222	0.001
$\lambda_{b}$	11.029	0.827	0.688	0.572	0.000	6.366	0.530	0.701	0.537	0.000
ε	−232.690	−0.159	−0.313	−0.241	0.000	−415.027	−0.572	−0.675	−0.704	0.000
CSAR	−0.413	−0.241	−0.105	−0.278	0.000	−0.621	−0.361	0.386	−0.293	0.000
$R^{2}$	0.742					0.796				
F	87.332					182.988				
Number	221					241				

B = unstandardized coefficient. β = standardized coefficient, Sig. = Significant level, EX = regression model excluded the variable.

**Table 3:** The step-wise multiple regression results of the SUHI prediction model.

Variables	LCZs 1–5					Mixed LCZ			
	Coefficient				Sig.	Coefficient			Sig.
	B	β	Partial Correlation			B	β	Partial Correlation	
Constant	235.605				0.000	405.985			0.000
α	−15.952	−0.216	−0.310		0.000	EX	EX	EX	EX
SVF	4.721	0.397	0.305		0.000	EX	EX	EX	EX
$\lambda_{v}$	−5.281	−0.413	−0.411		0.000	−5.749	−0.519	−0.611	0.000
FAR	−0.373	−0.333	−0.384		0.000	−0.303	−0.236	−0.193	0.007
$\lambda_{b}$	10.988	0.859	0.590		0.000	5.889	0.486	0.495	0.000
ε	−240.539	−0.184	−0.275		0.000	−415.054	−0.585	−0.714	0.000
CSAR	−0.447	−0.278	−0.310		0.000	−0.685	−0.410	−0.320	0.000
$R^{2}$	0.737					0.790			
F	70.327					146.288			
Number	184					201			

B = unstandardized coefficient, β = standardized coefficient, Sig. = Significant level, EX = regression model excluded the variable.
